# Nonfood GM Crops in Tropical Regions: A Reasonable Way to Promote the Technology for Increased Agricultural Sustainability

**DOI:** 10.1002/gch2.201800010

**Published:** 2018-05-29

**Authors:** Jean Carlos Cardoso

**Affiliations:** ^1^ Department of Biotechnology Plant and Animal Production Centro de Ciências Agrárias Universidade Federal de São Carlos Rod. Anhanguera, km 174 CEP13600‐970 Araras SP Brazil

**Keywords:** agriculture land expansion, biofuel and woody application, conservation of biodiversity, GM food x nonfood, regulatory keys

## Abstract

Modifying a plant genetically is the most remarkable technology developed for agriculture production; however, the use of genetically modified (GM) food crops has raised concerns regarding their impact on the human health and environment. Nevertheless, the nonfood GM crops, such as for biofuel and wood production, can be a solution to increase the yield and avoid deforestation promoted by the expansion of cultivable land. Thus, the biosafety regulatory framework of different countries can be revised and studied on a case‐by‐case basis, considering the rapid release of nonfood GM uses for the development of a more sustainable agriculture, particularly in tropical regions.

Transgenic or genetically modified (GM) plants are one of the most remarkable discoveries that could be transformed into a useful technology for agriculture and society. The transfer of genes, from one species to another, using the mechanism of infection of *Agrobacterium* or by direct transformation, has revolutionized the field of breeding and biotechnology of crop plants. The GM technology offers novel application of biotechnology in plant breeding, generating an essential tool to create new and improved varieties of cultivars and hybrids, allowing the transfer of only one gene of interest from any organism to a plant, overcoming limitations imposed by reproductive barriers with conventional breeding. Since its advent, several transgenic crops with a variety of traits including insect resistance, herbicide tolerance, environmental stress tolerance, and nutritionally enhanced cultivars for agronomic species have been developed.^1^ Despite this, some concerns regarding potential environmental safety and impact on human health have restricted to the consumption of transgenic crop varieties^2^ and limited the widespread application of the technique for the real purposes that were first presumed to benefit and accelerate the plant breeding process and resolve concerns related to achieving sustainable agriculture. Most of the questions can easily be answered through proper scientific investigations. However, the concerns regarding potential contamination by GM pollen of transgenic plants to nontransgenic crops, the unsubstantiated negative acceptance about the consumption of GM food crops in many countries,^3^ and the requirement of regulatory processes for the release of GM plants to the market, remain significant obstacles to further expansion of the GM crops. Nevertheless, society has longed for the sustainable development of agriculture, such as natural resources (mainly water and fertilizer) and pesticides reduction. In this context, industrial‐agriculture production of biofuels from sugarcane (*Saccharum × officinarum*) and oil palm (*Elaeis* sp.) and also for timber (*Pinus* sp.; *Eucalyptus* sp.) are highly dependent on the increased sustainability of agriculture to meet the growing global demands of these products and aim to obtain clean fuels and reduce CO_2_ emissions. In addition, these non‐food GM crops are large‐scale cultivated, mostly geographically distant to wild correlated species; this solves some of the cultural and environmental concerns about GM varieties. The complete or partial nonuse of GM products for food and the low environmental risks of these species offer their cultivation in regions where there are no‐wild relatives or that non‐GM cultivated varieties have a very low capacity of hybridization with a wild relative. For instance, most species of genus *Saccharum* (sugarcane) are from New Guinea and Asia and, in Brazil (South America), it is primarily cultivated using non‐GM varieties, which could hardly bloom in these cultivation conditions with very low viability of its pollen grains; thus this reduces the risks associated with contamination from GM pollen.^4^ Other production regions are Asia and Africa, where *Saccharum* wild‐type species occur more frequently, could also be benefited with transgenic GM sugarcane by using only similar varieties with reproductive limitations.

The significant challenges for GM sugarcane production are increased resistance to bacterial and virus diseases due to typical vegetative propagation and increased mechanized harvest that leads to considerable contamination by systemic diseases. Furthermore, abiotic factors, such as water stress and salinity resistance, could expand the cultivation in the regions with low biodiversity, instead of expansion of the plants in tropical forests areas^5^ (**Figure** **1**). Thus, the GM plants with salinity stress resistance may allow the use of nonpotable water for irrigation, increasing liquid residues from the sugarcane industry and also allow the use of domestic sewage for irrigation. Notably, the use of domestic sewage for irrigation could be achieved with the GM crops resistant to salinity stress not only for sugarcane but also for other nonfood crops. This is possible because most of these crops are cultivated in tropical regions at marginal areas of urban territories of São Paulo State, Brazil (Figure 1), where most of these types of residues are disposed at low cost and represent an economic and environmental problem for the sustainability of urban and periurban regions of cities.^6^

**Figure 1:**
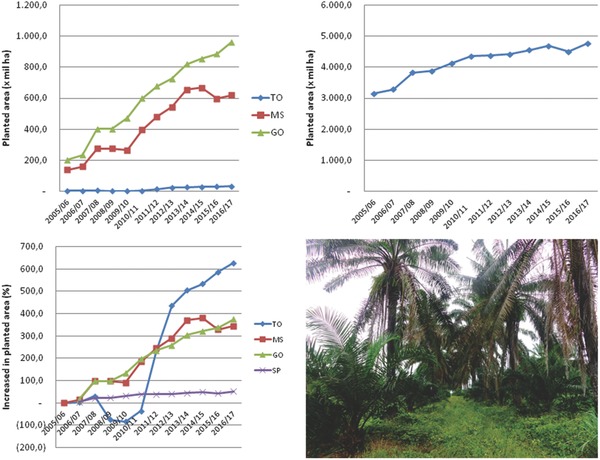
Total area and percentage increase in the planted area for sugarcane in Brazil regions (graphics) and the expansion of oil palm in Amazon forests (photo: original photo kindly provided by Prof. Douglas R. Bizari). Regions of Brazil: Tocantins (TO), Mato Grosso do Sul (MS), Goias (GO), São Paulo (SP). Source of datas (CONAB, 2017).

Furthermore, for oil palm, water deficiency is considered the primary agroclimatic limitation for the expansion of culture for biodiesel production. Consequently, the overexploitation of this crop is now leading to the deforestation, pollution, and loss to the biodiversity of several endangered species in the tropical forests in South and Central America, Asia, and Africa.^7^ Thus, the development of GM oil palm with resistance to water stress and salinity could result in the expansion of the plant for other cultivated or deforested areas with lower rain regimes than the equatorial tropical forests, e.g., Amazon, maintaining the high oil productivity through this variety and avoiding further deforestations in these habitats with high biodiversity. Therefore, it is important to point that the varieties used for similar purposes (biodiesel production), and also be extended to food crops, such as soybean (*Glycine max*) are in continuous GM cultivar releasing, as recently Intacta RR2 PRO soybean variety, that has received approval using GM technology.^8^

For wood plants (*Eucalyptus* and *Pinus*), the genetic transformation could also help in accelerating the growth and improving the durability of wood in the postharvest operations by not only improving the wood crops but also introducing the GM varieties for wood durability similar to that of noble woods tree wild species used illegaly. Besides this, noble woods wild species suffer from the illegal extraction and deforestation, reducing biodiversity in the tropical forests.^9^ Therefore, the GM wood species can control unlawful logging of wild vegetation adopted by the lucrative illegal timber industry.

The incorporation of the GM nonfood crops on a large scale in agriculture to sustain the feedstock requirements for bioenergy and furniture industry could lead to a new era of sustainable agriculture, particularly when the production and harvested area are rapidly expanding. For instance, total sugarcane area in Brazil increased 500 thousand hectares only in 2017, with to 3.5 to 6.3‐fold increase in cultivated area of Goias (GO), Mato Grosso do Sul (MS), and Tocantins (TO) states in Brazil during the last ten years (2006–2016)^10^ (Figure 1) and notably in most of Pantanal, Brazilian wild savanna, and with transitions of Amazon forest areas. Oil palm (*Elaeis* sp.) follows the similar increasing trend in the area for oil production and also reported a twofold increase in planted area between 2006 and 2013 in Brazil.^11^ Furthermore, some concerns in the regulatory framework of different countries could be revised for these nonfood crops, particularly for tropical regions, where most of these cultivable areas are located in the centers of biodiversity, and the release of GM varieties may contribute toward increased sustainability. Many debates have been undertaken about the potential risks and benefits of the GM food crops. Although it was necessary, now it is time for science to respond with ethics to the health and environmental safety concerns of these GM food crops. Moreover, rapid case‐by‐case studies are required on the regulation of the GM crops, as in tropical regions, the delayed release of some of the GM varieties, those used for biofuel and timber production, could lead to further economical (high cost of biofuels and wood) and, environmental (deforestation and biodiversity) losses. There are very low chances of impact and risk of these GM crops to human health and reproductive contamination of wild plant species, as they are mostly non‐native in the regions where they are cultivated efficiently. The release of GM variety should be accompanied by legislation prohibiting the use of these varieties for food purposes, in case of dual‐purpose varieties (food and nonfood), such as sugarcane and oil palm, as the release for food purpose requires more investigation.

There is no doubt that the GM varieties currently released on the market represent a major advance in plant breeding technology, but most of the released technologies represent mostly purely economic gains such as pest and herbicide resistance. In addition, many of the nonfood crops of major agricultural importance still do not benefit from these technologies because they follow similar legislation aspects of food crops GM varieties. These shortcomings have hampered aspects related to the conservation of the tropical forests, currently used for the cultivation of these species, as well as the use of transgenic for broader objectives, especially those related to abiotic factors, such as the development of the GM cultivars for conditions with water limitations or use of sewage water in the cultivation of these species, aiming energy and wood production.

## Conflict of Interest

The authors declare no conflict of interest.
